# Student-teacher agreement on personality traits and social competencies during early adolescence: A longitudinal study

**DOI:** 10.1371/journal.pone.0351394

**Published:** 2026-06-16

**Authors:** Marek Blatný, Veronika Koutná, Martin Jelínek, Petr Květon, Alexander T. Vazsonyi

**Affiliations:** 1 Department of Psychology, Faculty of Arts, Masaryk University, Brno, Czech Republic; 2 Department of Family Sciences, University of Kentucky, Lexington, Kentucky, United States of America; Prague University of Economics and Business: Vysoka Skola Ekonomicka v Praze, CZECHIA

## Abstract

The present study sought to validate existing knowledge on inter-rater agreement on personality traits and social competencies. Based on longitudinal data, the study aimed to determine whether the agreement between student and teacher reports changes over time. The research sample consisted of a total of 296 early adolescents (181 females, 115 males, average age in the first wave of the two-year study period was 12.37 years). The personality traits and social competencies of students were rated by class teachers (N = 28, 75% females). Personality traits were measured by the Big Five Inventory and social competencies by the Social Skills Improvement System. The main findings of the study are as follows: 1) the highest agreement between student and teacher ratings of personality traits and social competencies was found for characteristics that were high in observability; 2) there was greater agreement between student and teacher ratings of social competencies for girls; 3) high temporal stability was found for personality traits and social competencies for both students and teachers; there were no substantial changes in the agreement of student and teacher ratings over time. Attention to hidden or difficult-to-assess student characteristics may prevent developmental, school, and social/interpersonal problems in adolescents.

## Introduction

Inter-rater agreement on personality characteristics has received much attention in psychological research, as it helps establish the reliability and validity of personality assessments. Moreover, the degree of agreement between self-ratings and those of other observers has significant implications for various aspects of a person’s life. Low levels of self-observer agreement may indicate a discrepancy between an individual’s self-perception and the perceptions of others, and this mismatch could lead to misunderstandings and difficulties in forming and maintaining relationships [1]. Thus, for psychological research, it is particularly important to study the personality characteristics for which self-perceptions and those of others are most discrepant and to look for the causes and consequences of these discrepancies.

### Personality

Previous research on inter-rater agreement has focused primarily on personality traits in the context of the Five-Factor Model of personality [2–6]. In their seminal work, John and Robins [4] found that inter-rater agreement is associated with the content domain of the Five-Factor Model (FFM), observability, and social desirability. In addition, they focused on the differences between two types of inter-rater agreement: peer-peer agreement and self-peer agreement. They found that more observable and less evaluative traits such as Extraversion elicited higher inter-rater agreement. Self-peer agreement was lower than peer-peer agreement, but this effect was only for traits high in evaluativeness; for neutral traits, self-peer agreement was as high as peer-peer agreement.

Vazire [6] formulated the Self-Other Knowledge Asymmetry (SOKA) Model. According to this model, self-evaluation is more accurate than the judgment of others for traits with low observability (e.g., Neuroticism), whereas the judgement of others is more accurate than self-evaluation for traits with high evaluativeness (e.g., intellect). Trait observability is associated with informational asymmetry. The self has access to its own thoughts and feelings, which are inaccessible to the outside observer; others, on the other hand, have a better physical perspective on observable behavior. Trait evaluativeness is associated with motivational asymmetry. The main motivational difference between self-perception and the perception of others is the degree of ego involvement. Self-judgments of a highly evaluative trait are biased by various self-serving biases, whereas the judgments of others, who are not emotionally involved, may therefore be closer to reality.

An important issue with regard to inter-rater agreement is the acquaintance of the rater and the person being rated. In their meta-analysis, Connelly and Ones [2] found not only that increased frequency of interactions with the target persons increases the accuracy of their judgement of personality traits, but that a close personal proximity of informants to the target person is necessary to substantially increase the accuracy of their personality judgements, particularly for traits with low observability.

Research suggests that self-perceptions tend to be less accurate during adolescence and early adulthood, with individuals overestimating their positive qualities and underestimating their negative qualities. As individuals mature, self-perceptions become more aligned with observer perceptions, resulting in higher self-observer agreement [7,8].

### Social competencies

In recent years, in addition to personality traits, attention has also been paid to agreement in the assessment of social competencies in childhood and adolescence using behavioral rating scales with multiple informants (e.g., parents, teachers or classmates). However, there seems little agreement in this area, which accords with the low inter-rater agreement found in various research with children and adolescents [9]. In their meta-analysis of agreement on social competence, Renk and Phares [10] reported that agreement in informant pairs including child/adolescent self-report (e.g., teacher-student) is lower when compared to other informant pairs (e.g., teacher-parent). These findings are consistent with Gresham et al. [11] who found a similar level of teacher-student agreement, with the highest agreement for the Responsibility and Engagement competencies and the lowest agreement for the Communication and Empathy competencies.

These cross-informant discrepancies in perceptions of social competence do not necessarily imply measurement error or lack of validity. Several factors may influence the level of agreement, including observability and willingness to report [12]. The level of cross-informant agreement also varies depending on the age of the child. For example, Zsolnai and Kasik [13] found a decreasing level of teacher-student agreement on social skills between 7 and 11-year-olds, while Sørlie et al. [14] found increasing teacher-student cross-informant correlation between grades 8 and 10.

Most of the evidence on the development of social competence stems from studies of preschoolers and younger school-age children. These studies show that developmental trends may depend on the child’s sex and on the informant. Girls tend to be rated higher on social competencies in the ratings of teachers as early as in kindergarten, but also later in life [13–15]. Boys, on the other hand, were found to have higher levels of individual differences in social competence than girls [15]. Hajovsky et al. [15] described a linear decline in competence for boys and stagnation/stability for girls between kindergarten and grade 6 in the reports of teachers. However, the authors add that boys do not necessarily exhibit a decline in competence, but rather they fall short of the standards set by girls. Sørlie et al. [14] found that social competencies are established earlier in development and remain stable during adolescence.

### Objectives of the study

Research on the level of inter-rater agreement, particularly the SOKA model (Vazire, 2010), has highlighted the significant role of the Self in processing information about oneself. Markus [16] introduced the concept of the Self as a system of cognitive schemas. According to Markus, people have developed a schema for their important traits; thus, they are “schematic” for certain areas of personal characteristics, while for those areas of personal characteristics for which they have not developed a schema—and which are therefore not part of their self-concept—they are “aschematic.” For an individual to be able to create such a schema, the characteristic in question must not only be descriptive but also important to them. Previous research has been based primarily on self-descriptive scales and has not taken into account the subjective importance of the behavioral, cognitive, and emotional characteristics being assessed. This study aims to further investigate whether what is accessible only to the self—the subjective importance of a personal characteristic—can be observed by an external rater through the behavior of the person being evaluated.

The objective of the present study is therefore to validate existing knowledge on the effect of the observability of personality traits on the level of inter-rater agreement and to extend it to social competencies with the inclusion of a scale assessing the “importance” of social competencies. The assumption is that social competencies such as Cooperation and Engagement are highly visible compared to more private ones such as Empathy and Self-Control [11,17], and that the agreement will be higher for more visible competencies. It can also be assumed that the level of a given social skill will be more readily apparent in the child’s behavior than the child’s perception of its subjective importance, and therefore, agreement on the rating of level will be higher than agreement on the rating of the importance of a competence.

On the basis of longitudinal data, the study aims to determine whether agreement between self- and teacher reports changes over time and whether teachers are able to identify developmental trends in personality traits and social competencies. Research suggests that inter-rater agreement on personality traits tends to be lower during adolescence compared to middle adulthood [8] and the level of teacher-student agreement on social skills is reported to be lower for older school-age children compared to younger school-age children [13]. Therefore, it was expected that the level of student-teacher agreement on personality traits and social competencies would be generally low and stable over time.

## Method

### Ethics statements

The study was approved by the University of Kentucky Institutional Review Board (14–0623-P4S) and by the Board of the Institute of Psychology, Czech Academy of Sciences. Written informed consent was obtained from parents of all participants in the study.

### Procedure

The current longitudinal study is part of the Brno Longitudinal Study of Youth (BLSY), an accelerated longitudinal study that followed two cohorts of youth (at grades 6 and 7), aged 12 and 13 years at baseline. The convenience sample was recruited from nine schools in Brno, a medium-sized city in the Czech Republic, which were selected on the basis of the school’s interest and willingness to participate. The recruitment for this study took place from 10/10/2014–11/11/2014. The study received University IRB approval and followed ethical guidelines; written informed consent was obtained from parents of all participants in the study.

The study analyzed data from four waves of data collection. The first wave of data collection took place in December 2014, followed by subsequent waves at six-month intervals. The data were collected in group settings in classrooms using a paper-and-pencil questionnaire (taking around 90 minutes to complete), which was administered in a controlled school environment by trained administrators. The students and their class teachers completed their questionnaires concurrently. Teachers received financial compensation for rating their students.

The scale scores were computed as the average response to items, where there were no more than two missing responses for a given scale. During the first wave of data collection, valid data were obtained from 453 students (the total scores of the observed variables were successfully computed for all students and each of them received an assessment from their class teacher). Valid data was obtained from 490 students in the second wave, from 462 students in the third wave, and from 468 students in the fourth wave. Due to the longitudinal nature of the study, only data from those students who participated in all four waves of data collection and received teacher ratings were included in the subsequent analyses.

### Research sample

The research sample consisted of a total of 296 participants (181 females, 115 males). The average age of the participants in the first wave of data collection was 12.37 years (SD = 0.63), who were attending either first grade (N = 57) or second grade (N = 39) in the case of Gymnasiums or high schools, or sixth grade (N = 102) or seventh grade (N = 98) in the case of primary schools or middle schools. The personality traits and social competencies of students were assessed by their class teachers (form tutors) (N = 28, 75% females).

### Instruments

The Big Five Inventory (BFI; [18]) assessed five basic personality traits, based on 44 items rated on a five-point response scale (ranging from 1 to 5). In addition to rating their own traits, the respondents were also rated by their class teachers. The internal consistencies of all dimensions were satisfactory (McDonald’s *ω* > 0.70) with the exception of self-reported Extraversion (McDonald’s *ω* = 0.58) and Agreeableness (McDonald’s *ω* = 0.69).

The Social Skills Improvement System (SSIS; [19]) was used to assess behavioral and interpersonal skill difficulties, screen for problem behaviors, and identify students at risk of social behavior difficulties and poor academic performance. In this study, the SSIS scales assessing social competencies were utilized (Communication, Cooperation, Assertion, Responsibility, Empathy, Engagement, and Self-control. Each item was rated for the truthfulness of the statement (on a four-point scale from 1 to 4) and the importance of the skill (on a three-point scale from 1 to 3). In addition to rating their own competencies, respondents were also rated by their class teachers. The internal consistency of the scales was satisfactory (McDonald’s *ω* > 0.70), although eight scales had slightly lower internal consistency (McDonald’s *ω* > 0.60; the lowest level was the Importance Responsibility scale for teachers with *ω* = 0.62). Internal consistency was assessed in the first wave of measurement.

### Data analysis

Pearson correlations were used for assessing the agreement between the self-ratings and the teacher ratings. In the case of personality traits, the mean values for each dimension were also compared using paired t-tests. However, this was not possible for social competencies due to the differences in the content of the items used for self-ratings and teacher ratings. The stability of the self-ratings and teacher ratings was examined using Pearson correlations, and changes in levels were assessed using General Linear Model for repeated measures. A procedure based on Fisher’s z transformation was employed to calculate averaged correlation coefficients and Cohen’s *d*. All analyses were conducted separately for girls and boys, and sex was considered as a between-subject factor in the analysis of variance. Effect sizes were interpreted following the recommendation by Funder and Ozer [20]: *r* > 0.10 (*d* > 0.20; *η*_*p*_^*2*^ > 0.01) indicated a small effect size, *r* > 0.20 (*d* > 0.41; *η*_*p*_^*2*^ > 0.04) indicated a medium effect size, and *r* > 0.30 (*d* > 0.63; *η*_*p*_^*2*^ > 0.09) indicated a large effect size.

## Results

### Agreement between self-ratings and teacher ratings – personality traits

From the correlation coefficients between the self-ratings and teacher ratings of personality traits among boys (see Table 1) and girls (Table 2), it can be concluded that the agreement between ratings is generally not high. The highest values across all measurement waves for both boys and girls were observed for Extraversion, as is evident from the average correlation coefficient shown in Table 3. Similarly, moderate agreement was observed for Openness among both boys and girls, for which average correlation coefficients for self-other agreement were almost identical. In contrast to the aforementioned traits, differences were found between girls and boys in the level of agreement for Conscientiousness, where the coefficients for girls were statistically significant and higher than 0.2 in all waves.

**Table 1:** The relationship between self-rating and teacher-rating of personality traits in males.

			*Mean*	*SD*	*t*	*p*	*d*	*r*	*p*
*Wave 1*	E	(S)	3.47	0.45	3.01	0.003	**0.28**	**0.34**	0.000
		(T)	3.24	0.86					
	A	(S)	3.65	0.51	0.72	0.476	0.07	0.12	0.222
		(T)	3.60	0.60					
	C	(S)	3.35	0.60	1.16	0.249	0.11	**0.20**	0.030
		(T)	3.25	0.84					
	N	(S)	2.72	0.63	1.66	0.101	0.15	0.03	0.756
		(T)	2.58	0.67					
	O	(S)	3.42	0.64	3.03	0.003	**0.28**	**0.30**	0.001
		(T)	3.20	0.67					
*Wave 2*	E	(S)	3.43	0.58	3.64	0.000	**0.34**	**0.40**	0.000
		(T)	3.16	0.84					
	A	(S)	3.58	0.55	−1.26	0.211	−0.12	0.14	0.136
		(T)	3.67	0.56					
	C	(S)	3.27	0.62	0.94	0.348	0.09	**0.25**	0.008
		(T)	3.19	0.88					
	N	(S)	2.75	0.67	1.98	0.050	**0.19**	−0.06	0.527
		(T)	2.57	0.67					
	O	(S)	3.35	0.56	2.14	0.034	**0.20**	0.18	0.057
		(T)	3.20	0.63					
*Wave 3*	E	(S)	3.48	0.57	4.19	0.000	**0.39**	**0.49**	0.000
		(T)	3.18	0.86					
	A	(S)	3.63	0.47	−0.051	0.959	−0.01	**0.20**	0.033
		(T)	3.63	0.60					
	C	(S)	3.30	0.63	1.36	0.175	0.13	0.07	0.432
		(T)	3.16	0.93					
	N	(S)	2.62	0.63	−0.48	0.631	−0.05	−0.06	0.530
		(T)	2.66	0.69					
	O	(S)	3.38	0.59	2.42	0.017	**0.23**	**0.21**	0.023
		(T)	3.20	0.69					
*Wave 4*	E	(S)	3.34	0.56	2.15	0.034	**0.20**	**0.43**	0.000
		(T)	3.18	0.83					
	A	(S)	3.49	0.59	−2.17	0.032	**−0.20**	**0.22**	0.017
		(T)	3.64	0.56					
	C	(S)	3.28	0.60	1.30	0.198	0.12	0.17	0.064
		(T)	3.16	0.89					
	N	(S)	2.74	0.70	1.51	0.133	0.14	0.12	0.186
		(T)	2.61	0.69					
	O	(S)	3.29	0.65	1.18	0.240	0.11	**0.21**	0.023
		(T)	3.20	0.61					

Statistically significant statistics (*p* < 0.05) are bolded. (S) = student’s self-rating; (T) = teacher-rating. E = Extraversion, A = Agreeableness, C = Conscientiousness, N = Neuroticism, O = Openness.

**Table 2:** The relationship between self-rating and teacher-rating of personality traits in females.

			*Mean*	*SD*	*t*	*p*	*d*	*r*	*p*
*Wave 1*	E	(S)	3.60	0.61	6.52	0.000	**0.49**	**0.44**	0.000
		(T)	3.18	0.93					
	A	(S)	3.88	0.49	0.92	0.360	0.07	**0.15**	0.044
		(T)	3.84	0.51					
	C	(S)	3.49	0.56	−0.80	0.427	−0.06	**0.24**	0.001
		(T)	3.54	0.86					
	N	(S)	2.93	0.73	4.66	0.000	**0.35**	**0.19**	0.011
		(T)	2.62	0.67					
	O	(S)	3.62	0.56	5.85	0.000	**0.44**	**0.24**	0.001
		(T)	3.28	0.68					
*Wave 2*	E	(S)	3.58	0.67	5.18	0.000	**0.39**	**0.35**	0.000
		(T)	3.24	0.86					
	A	(S)	3.81	0.52	−0.38	0.702	−0.03	**0.16**	0.037
		(T)	3.83	0.51					
	C	(S)	3.45	0.66	−1.81	0.072	−0.14	**0.37**	0.000
		(T)	3.56	0.80					
	N	(S)	2.92	0.77	5.39	0.000	**0.40**	0.11	0.138
		(T)	2.54	0.62					
	O	(S)	3.56	0.58	3.67	0.000	**0.27**	0.11	0.141
		(T)	3.34	0.62					
*Wave 3*	E	(S)	3.48	0.70	5.46	0.000	**0.41**	**0.44**	0.000
		(T)	3.14	0.89					
	A	(S)	3.81	0.53	−0.23	0.817	−0.02	**0.18**	0.017
		(T)	3.82	0.55					
	C	(S)	3.42	0.61	−2.18	0.036	**−0.16**	**0.32**	0.000
		(T)	3.55	0.82					
	N	(S)	3.02	0.79	4.23	0.000	**0.31**	0.03	0.659
		(T)	2.71	0.64					
	O	(S)	3.55	0.57	2.76	0.006	**0.21**	**0.25**	0.001
		(T)	3.40	0.64					
*Wave 4*	E	(S)	3.47	0.67	5.83	0.000	**0.43**	**0.43**	0.000
		(T)	3.11	0.86					
	A	(S)	3.75	0.55	−1.39	0.193	−0.10	**0.15**	0.050
		(T)	3.82	0.54					
	C	(S)	3.41	0.63	−2.25	0.026	**−0.17**	**0.23**	0.002
		(T)	3.56	0.81					
	N	(S)	3.07	0.81	4.64	0.000	**0.35**	0.00	0.974
		(T)	2.71	0.64					
	O	(S)	3.56	0.62	3.27	0.001	**0.24**	**0.30**	0.000
		(T)	3.38	0.61					

Statistically significant statistics (*p* < 0.05) are bolded. (S) = student’s self-rating; (T) = teacher-rating. E = Extraversion, A = Agreeableness, C = Conscientiousness, N = Neuroticism, O = Openness.

**Table 3:** Averaged agreement between self-rating and teacher-rating of personality traits across all waves.

	*Males*		*Females*	
	*Averaged r*	*Averaged d*	*Averaged r*	*Averaged d*
Extraversion	0.42	0.30	0.42	0.42
Agreeableness	0.17	−0.06	0.16	−0.02
Conscientiousness	0.18	0.11	0.29	−0.13
Neuroticism	0.01	0.11	0.08	0.35
Openness	0.23	0.20	0.22	0.29

In terms of mean levels (Cohen’s *d*), the differences were in the category of at least small effect sizes for Extraversion and Openness in both girls and boys, and for Neuroticism only in girls, as can be seen in Table 3. In all of the aforementioned cases, teachers consistently reported lower values of the traits than the students’ self-ratings. This difference was most pronounced for Extraversion, which was observed in all waves for both girls and boys, with the overall effect size within the range of a medium effect. There was a similarly marked difference for Neuroticism in girls, for which girls’ self-ratings were consistently higher than the teacher ratings in all waves, although this effect was not found for boys.

### Agreement between self-ratings and teacher ratings – social competencies

In contrast to the analysis of personality traits, no mean level comparisons were made between self-ratings and teacher ratings, due to the differences in the composition of items. Therefore, Tables 4 and 5 only summarize the data on the agreement between self-ratings and teacher ratings in the domain of social competencies and their importance. All the data indicate a low level of agreement between self-ratings and teacher ratings in the area of social competencies. In terms of importance, only a few significant correlation coefficients were found, apart from in the fourth wave of measurement for boys, in which there was a significant agreement in the rating of four skills. Although the agreement between self-ratings and teacher ratings for social competencies overall can be considered relatively low, Cooperation and Engagement stand out as exceptions. For these competencies, relationships between the ratings were consistently found for boys and girls, ranging from a small to medium effect size in boys and a medium to large effect size in girls.

**Table 4:** The relationship between self-rating and teacher-rating of social skills in males.

			*Social skills*				*Social skills – importance*			
			*Mean*	*SD*	*r*	*p*	*Mean*	*SD*	*r*	*p*
*W1*	Comm	(S)	3.05	0.44	0.07	0.454	2.25	0.40	−0.09	0.327
		(T)	2.91	0.39			1.99	0.23		
	Coop	(S)	2.90	0.48	0.14	0.139	2.29	0.39	−0.08	0.412
		(T)	2.83	0.49			2.21	0.31		
	Assert	(S)	2.77	0.50	0.10	0.296	2.10	0.37	−0.04	0.650
		(T)	2.68	0.41			1.91	0.24		
	Resp	(S)	2.93	0.40	0.04	0.702	2.38	0.33	−0.03	0.743
		(T)	2.81	0.44			2.08	0.27		
	Emp	(S)	2.98	0.54	0.03	0.735	2.31	0.41	−0.06	0.556
		(T)	2.66	0.46			1.86	0.27		
	Eng	(S)	3.01	0.50	0.16	0.080	2.18	0.39	−0.02	0.820
		(T)	2.70	0.45			1.83	0.34		
	Slf-Ctrl	(S)	2.70	0.48	−0.08	0.407	2.16	0.40	−0.02	0.866
		(T)	2.74	0.46			2.02	0.25		
*W2*	Comm	(S)	3.06	0.46	0.09	0.347	2.20	0.46	0.13	0.160
		(T)	2.92	0.30			2.05	0.22		
	Coop	(S)	2.86	0.45	**0.20**	0.034	2.23	0.39	0.04	0.689
		(T)	2.87	0.46			2.21	0.28		
	Assert	(S)	2.73	0.43	0.15	0.101	2.07	0.40	0.13	0.175
		(T)	2.69	0.38			1.94	0.26		
	Resp	(S)	2.91	0.39	0.02	0.831	2.26	0.39	−0.01	0.908
		(T)	2.91	0.42			2.18	0.30		
	Emp	(S)	2.94	0.48	0.13	0.160	2.22	0.41	−0.07	0.449
		(T)	2.73	0.38			1.95	0.31		
	Eng	(S)	2.97	0.46	0.13	0.155	2.13	0.42	0.13	0.165
		(T)	2.70	0.41			1.94	0.25		
	Slf-Ctrl	(S)	2.58	0.56	0.06	0.543	2.03	0.47	0.12	0.222
		(T)	2.81	0.38			2.03	0.20		
*W3*	Comm	(S)	3.10	0.46	0.09	0.327	2.20	0.46	0.18	0.054
		(T)	2.90	0.34			1.98	0.25		
	Coop	(S)	2.92	0.43	**0.28**	0.002	2.15	0.42	−0.05	0.615
		(T)	2.81	0.53			2.17	0.34		
	Assert	(S)	2.77	0.43	0.14	0.149	2.02	0.42	0.07	0.436
		(T)	2.70	0.43			1.97	0.29		
	Resp	(S)	2.95	0.39	0.16	0.095	2.27	0.39	0.07	0.457
		(T)	2.89	0.44			2.11	0.27		
	Emp	(S)	2.98	0.49	0.16	0.095	2.20	0.43	0.13	0.163
		(T)	2.65	0.48			1.89	0.33		
	Eng	(S)	2.99	0.50	**0.23**	0.015	2.12	0.42	**0.29**	0.002
		(T)	2.65	0.45			1.84	0.35		
	Slf-Ctrl	(S)	2.68	0.55	0.09	0.358	2.07	0.47	0.17	0.067
		(T)	2.77	0.42			2.00	0.21		
*W4*	Comm	(S)	3.01	0.50	**0.19**	0.044	2.11	0.45	**0.25**	0.006
		(T)	2.88	0.33			1.90	0.27		
	Coop	(S)	2.87	0.43	**0.25**	0.008	2.13	0.38	0.02	0.870
		(T)	2.78	0.46			2.17	0.26		
	Assert	(S)	2.73	0.47	0.17	0.063	2.00	0.38	**0.31**	0.001
		(T)	2.71	0.39			1.95	0.21		
	Resp	(S)	2.94	0.40	0.08	0.388	2.20	0.37	−0.13	0.157
		(T)	2.88	0.44			2.14	0.25		
	Emp	(S)	2.90	0.52	**0.19**	0.039	2.09	0.39	**0.33**	0.000
		(T)	2.72	0.39			1.91	0.21		
	Eng	(S)	2.88	0.48	0.13	0.177	2.03	0.39	**0.23**	0.012
		(T)	2.67	0.41			1.91	0.20		
	Slf-Ctrl	(S)	2.71	0.55	0.13	0.164	2.04	0.43	0.16	0.083
		(T)	2.74	0.37			2.02	0.15		

Statistically significant statistics (*p* < 0.05) are bolded. (S) – student’s self-rating; (T) – teacher-rating. Comm = Communication, Coop = Cooperation, Assert = Assertion, Resp = Responsibility, Emp = Empathy, Eng = Engagement, Slf-Ctrl = Self-Control.

**Table 5:** The relationship between self-rating and teacher-rating of social skills in females.

			*Social skills*				*Social skills – importance*			
			*Mean*	*SD*	*r*	*p*	*Mean*	*SD*	*r*	*p*
*W1*	Comm	(S)	3.26	0.38	0.03	0.658	2.37	0.37	−0.09	0.228
		(T)	3.09	0.36			2.04	0.29		
	Coop	(S)	3.00	0.43	**0.19**	0.010	2.32	0.36	0.01	0.186
		(T)	3.07	0.45			2.17	0.32		
	Assert	(S)	2.88	0.44	0.12	0.100	2.21	0.37	−0.06	0.452
		(T)	2.73	0.47			2.00	0.34		
	Resp	(S)	2.99	0.41	0.06	0.439	2.38	0.36	−0.11	0.131
		(T)	3.11	0.38			2.13	0.30		
	Emp	(S)	3.32	0.42	0.18	0.118	2.50	0.34	−0.11	0.144
		(T)	2.96	0.37			1.97	0.36		
	Eng	(S)	3.03	0.43	**0.23**	0.001	2.19	0.38	−0.10	0.198
		(T)	2.77	0.57			1.95	0.37		
	Slf-Ctrl	(S)	2.73	0.52	**0.17**	0.026	2.20	0.42	0.04	0.620
		(T)	2.97	0.38			2.07	0.31		
*W2*	Comm	(S)	3.28	0.39	**0.19**	0.011	2.34	0.40	0.01	0.912
		(T)	3.04	0.30			2.04	0.24		
	Coop	(S)	3.01	0.41	**0.28**	0.000	2.25	0.39	0.10	0.181
		(T)	3.03	0.42			2.16	0.27		
	Assert	(S)	2.89	0.42	**0.17**	0.027	2.18	0.35	0.06	0.459
		(T)	2.73	0.40			1.93	0.24		
	Resp	(S)	3.02	0.39	**0.16**	0.028	2.34	0.36	0.00	0.979
		(T)	3.08	0.37			2.12	0.29		
	Emp	(S)	3.28	0.44	0.14	0.067	2.40	0.37	**−0.17**	0.026
		(T)	2.95	0.36			1.98	0.28		
	Eng	(S)	3.01	0.49	**0.25**	0.001	2.18	0.40	0.03	0.686
		(T)	2.77	0.49			1.96	0.25		
	Slf-Ctrl	(S)	2.71	0.53	**0.15**	0.040	2.18	0.45	−0.04	0.593
		(T)	2.98	0.34			2.01	0.22		
*W3*	Comm	(S)	3.23	0.39	**0.20**	0.007	2.28	0.38	0.14	0.058
		(T)	3.02	0.32			2.01	0.21		
	Coop	(S)	2.99	0.44	**0.33**	0.000	2.22	0.39	0.01	0.930
		(T)	3.03	0.40			2.12	0.30		
	Assert	(S)	2.90	0.43	**0.19**	0.010	2.17	0.37	0.05	0.509
		(T)	2.74	0.44			1.96	0.30		
	Resp	(S)	3.04	0.41	**0.28**	0.000	2.31	0.37	**0.15**	0.039
		(T)	3.08	0.34			2.09	0.25		
	Emp	(S)	3.27	0.45	0.06	0.400	2.37	0.40	−0.03	0.710
		(T)	2.97	0.34			1.93	0.29		
	Eng	(S)	2.95	0.52	**0.24**	0.001	2.09	0.42	0.05	0.547
		(T)	2.76	0.48			1.89	0.30		
	Slf-Ctrl	(S)	2.78	0.52	**0.18**	0.015	2.15	0.39	**0.20**	0.006
		(T)	2.97	0.33			2.01	0.18		
*W4*	Comm	(S)	3.26	0.39	**0.24**	0.001	2.29	0.41	0.07	0.339
		(T)	3.02	0.33			1.99	0.24		
	Coop	(S)	3.00	0.43	**0.39**	0.000	2.22	0.38	−0.04	0.621
		(T)	3.05	0.41			2.15	0.28		
	Assert	(S)	2.94	0.40	**0.21**	0.005	2.11	0.36	−0.09	0.239
		(T)	2.74	0.44			1.96	0.18		
	Resp	(S)	3.05	0.39	**0.27**	0.000	2.27	0.37	−0.01	0.941
		(T)	3.08	0.36			2.11	0.26		
	Emp	(S)	3.30	0.42	0.13	0.075	2.34	0.39	−0.03	0.658
		(T)	2.99	0.37			1.94	0.26		
	Eng	(S)	2.95	0.48	**0.26**	0.000	2.06	0.40	0.04	0.587
		(T)	2.75	0.50			1.93	0.19		
	Slf-Ctrl	(S)	2.78	0.50	0.07	0.379	2.13	0.39	0.02	0.822
		(T)	2.96	0.37			2.02	0.13		

Statistically significant statistics (*p* < 0.05) are bolded. (S) – student’s self-rating; (T) – teacher-rating. Comm = Communication, Coop = Cooperation, Assert = Assertion, Resp = Responsibility, Emp = Empathy, Eng = Engagement, Slf-Ctrl = Self-Control.

Table 6 provides aggregated data for all four waves of measurement. It is evident that there is essentially only weak agreement between the self-ratings and teacher ratings, although it is stronger for Cooperation and Engagement, and more so for girls. With regard to the importance of these skills, there is virtually no agreement between the ratings.

**Table 6:** Averaged agreement between self-rating and teacher-rating of social skills across all waves.

	*Averaged r*			*Averaged r*	
*Social skills*	*Males*	*Females*	*Importance*	*Males*	*Females*
Communication	0.11	0.17	Communication	0.12	0.03
Cooperation	0.22	0.30	Cooperation	−0.02	0.04
Assertion	0.14	0.17	Assertion	0.12	−0.01
Responsibility	0.07	0.20	Responsibility	−0.03	0.01
Empathy	0.13	0.11	Empathy	0.09	−0.08
Engagement	0.16	0.25	Engagement	0.16	0.01
Self-Control	0.05	0.14	Self-Control	0.11	0.06

### Stability of self-ratings and teacher ratings – personality traits

The stability of personality trait ratings is generally high (Table 7). When comparing stability coefficients for teachers and students, the data clearly indicate that teacher ratings exhibit higher overall stability than self-ratings. With regard to differences between girls and boys, the only noticeable differences can be found for self-ratings. Among girls, the stability of the Extraversion and Neuroticism traits is slightly higher than for boys.

**Table 7:** Stability of self-rating and teacher-rating in consecutive waves (Pearson correlations) – personality traits.

	*Males*				*Females*			
*Self-rating*	*w1-w2*	*w2-w3*	*w3-w4*	*Avg*	*w1-w2*	*w2-w3*	*w3-w4*	*Avg*
Extraversion	0.57	0.57	0.57	0.57	0.72	0.76	0.79	0.76
Agreeableness	0.58	0.71	0.69	0.66	0.54	0.61	0.69	0.62
Conscientiousness	0.66	0.74	0.71	0.70	0.65	0.78	0.72	0.72
Neuroticism	0.60	0.68	0.52	0.60	0.70	0.69	0.76	0.72
Openness	0.53	0.70	0.66	0.63	0.66	0.72	0.74	0.71
*Teacher-rating*								
Extraversion	0.76	0.82	0.88	0.83	0.77	0.85	0.88	0.84
Agreeableness	0.67	0.79	0.77	0.75	0.71	0.74	0.80	0.75
Conscientiousness	0.73	0.85	0.84	0.81	0.79	0.79	0.83	0.81
Neuroticism	0.64	0.74	0.79	0.730	0.67	0.64	0.68	0.66
Openness	0.72	0.82	0.81	0.78	0.73	0.69	0.81	0.75

All coefficients are significant (p < 0.01).

**Fig 1:**
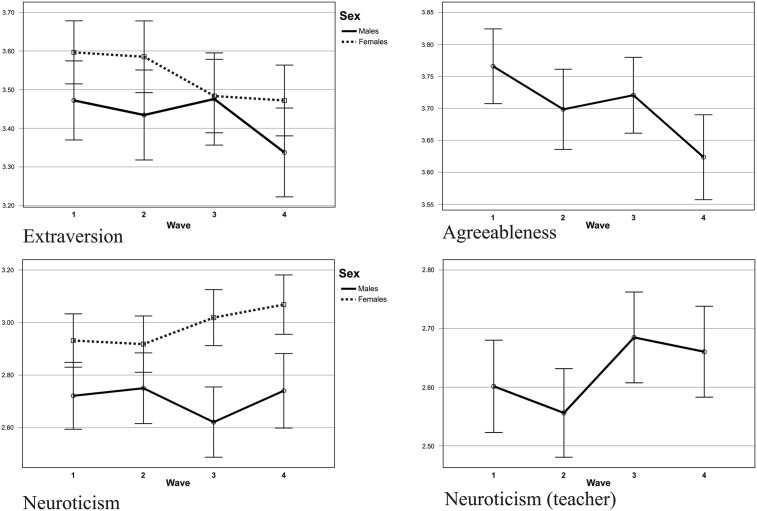
Differences in self-rating and teacher-rating based on time and sex – personality traits. Estimated marginal means and corresponding 95% confidence intervals are displayed.

Table 8 presents the results of the General Linear Model, which captures the changes in personality traits over time, taking into account the sex of the individuals rated. In the case of Extraversion, a significant change was found for this trait over time, which was further conditioned by the sex of the respondents. Fig 1 illustrates that while girls showed the most pronounced decline between the second and third measurement, boys experienced this shift between the third and fourth measurement. For Agreeableness, a relatively substantial change was observed over time, which was primarily driven by the difference between the levels measured in the first and final waves, with no interaction effect of sex. In the case of Neuroticism, an interaction effect of time and sex was found, with a gradual increase in this trait among girls, while for boys it remained relatively constant. From the perspective of teachers, there was a perceived difference in the level of Neuroticism in students between the first and second measurements when compared to the third and fourth measurements.

**Table 8:** Differences in self-rating and teacher-rating depending on time and sex – personality traits.

	*Wave*				Wave*sex			
*Self-rating*	*Wilk’s λ*	*F(3,292)*	*p*	*η*_*p*_^*2*^	*Wilk’s λ*	*F(3,292)*	*p*	*η*_*p*_^*2*^
Extraversion	0.95	4.73	0.003	**0.05**	0.97	2.72	0.045	**0.03**
Agreeableness	0.93	7.34	0.000	**0.07**	0.99	0.88	0.454	0.01
Conscientiousness	0.98	1.90	0.129	0.02	0.97	0.39	0.758	0.00
Neuroticism	0.98	1.99	0.115	0.02	0.96	3.91	0.009	**0.04**
Openness	0.98	2.30	0.077	0.02	0.99	1.08	0.359	0.01
*Teacher-rating*								
Extraversion	0.99	1.25	0.293	0.01	0.97	2.58	0.054	0.02
Agreeableness	0.99	0.64	0.591	0.01	0.99	0.61	0.608	0.01
Conscientiousness	1.00	0.42	0.741	0.00	0.99	0.89	0.474	0.01
Neuroticism	0.94	6.19	0.000	**0.06**	0.99	1.38	0.250	0.01
Openness	0.99	1.190	0.314	0.01	0.99	1.14	0.335	0.01

Statistically significant statistics (*p* < 0.05) are bolded. Due to a violation of the sphericity assumption, the results of Multivariate Tests are interpreted instead of Tests of Within-Subjects Effects.

### Stability of self-ratings and teacher-ratings – social competencies

The temporal stability of the self-rating of social competencies is generally comparable across different scales and typically ranges from 0.5 to 0.8 (see Table 9). Furthermore, neither the sex of the person rated nor the evaluator’s role (student vs. teacher) significantly influenced the level of stability. Regarding the stability of the rating of the importance of social competencies, the stability of the teacher rating of Self-Control deviates from the general pattern for both boys and girls.

**Table 9:** The stability of self-rating and teacher-rating in consecutive waves (Pearson correlations) – social skills and their importance.

*SOCIAL SKILLS*	*Males*				*Females*			
*Self-rating*	*w1-w2*	*w2-w3*	*w3-w4*	*Avg*	*w1-w2*	*w2-w3*	*w3-w4*	*Avg*
Communication	0.57	0.55	0.60	0.58	0.51	0.52	0.64	0.56
Cooperation	0.60	0.58	0.52	0.57	0.66	0.61	0.69	0.65
Assertion	0.52	0.63	0.63	0.60	0.63	0.66	0.69	0.66
Responsibility	0.57	0.61	0.54	0.58	0.65	0.63	0.66	0.65
Empathy	0.55	0.69	0.60	0.61	0.57	0.55	0.54	0.55
Engagement	0.58	0.68	0.59	0.62	0.57	0.660	0.77	0.68
Self-Control	0.38	0.51	0.54	0.48	0.62	0.63	0.67	0.64
*Teacher-rating*								
Communication	0.56	0.73	0.75	0.69	0.46	0.63	0.66	0.59
Cooperation	0.63	0.74	0.77	0.72	0.69	0.67	0.77	0.71
Assertion	0.51	0.61	0.72	0.62	0.69	0.68	0.72	0.70
Responsibility	0.54	0.63	0.71	0.63	0.47	0.65	0.73	0.63
Empathy	0.40	0.59	0.71	0.58	0.44	0.71	0.68	0.62
Engagement	0.590	0.75	0.75	0.71	0.70	0.78	0.75	0.75
Self-Control	0.537	0.62	0.72	0.63	0.55	0.72	0.74	0.67
***IMPORTANCE***	***Males***				***Females***			
***Self-rating***	***w1-w2***	***w2-w3***	***w3-w4***	***Avg***	***w1-w2***	***w2-w3***	***w3-w4***	***Avg***
Communication	0.49	0.64	0.58	0.57	0.60	0.66	0.62	0.63
Cooperation	0.63	0.61	0.64	0.63	0.65	0.62	0.66	0.64
Assertion	0.53	0.59	0.63	0.59	0.56	0.61	0.63	0.60
Responsibility	0.46	0.67	0.55	0.57	0.62	0.60	0.62	0.61
Empathy	0.47	0.64	0.64	0.59	0.47	0.56	0.59	0.54
Engagement	0.51	0.59	0.61	0.57	0.56	0.61	0.64	0.61
Self-Control	0.50	0.64	0.62	0.59	0.59	0.54	0.60	0.58
*Teacher-rating*								
Communication	0.36	0.66	0.45	0.50	0.24	0.66	0.50	0.48
Cooperation	0.51	0.62	0.71	0.62	0.49	0.60	0.65	0.58
Assertion	0.28	0.72	0.75	0.62	0.41	0.61	0.58	0.54
Responsibility	0.42	0.79	0.48	0.60	0.38	0.75	0.61	0.60
Empathy	0.41	0.63	0.61	0.56	0.42	0.65	0.63	0.58
Engagement	*0.18*	0.54	0.67	0.49	0.30	0.56	0.66	0.52
Self-Control	*−0.04*	0.36	0.64	0.35	*0.11*	0.29	0.30	0.23

All coefficients (with the exception of coefficients in italics) are significant (*p* < 0.01).

**Fig 2:**
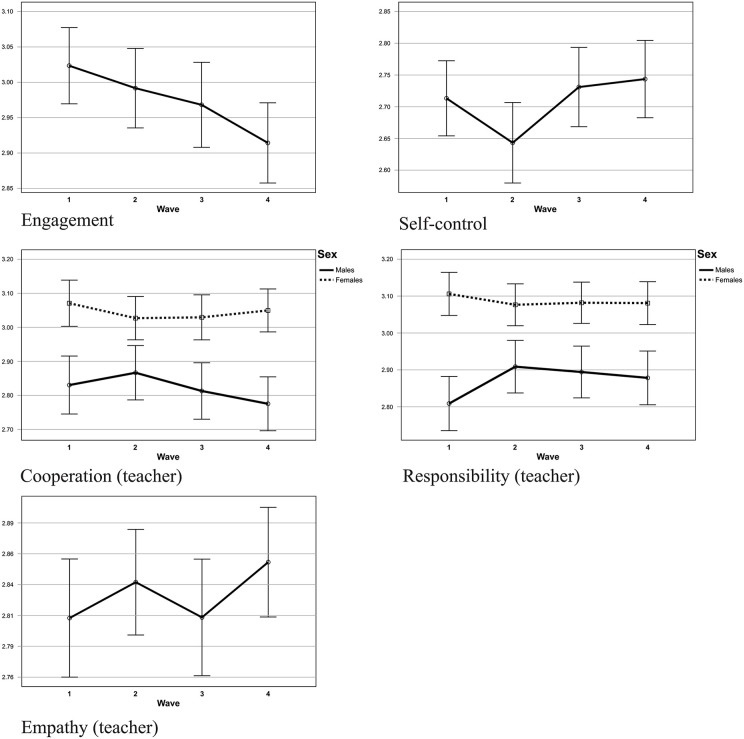
Differences in self-rating and teacher-rating based on time and sex – social skills. Estimated marginal means and corresponding 95% confidence intervals are displayed.

With regard to changes in the level of self-rating of social competencies (see Table 10 and Fig 2), the level of Engagement gradually decreases over time, regardless of sex. In the case of Self-Control, a significant decline was observed in the second wave of measurement. From the perspective of teachers, significant changes over time were found for Cooperation, Responsibility, and Empathy. For the first two aforementioned social competencies, an interaction effect of time and sex was identified, whereby teachers perceived both Cooperation and Responsibility to be higher in girls than in boys. In the case of Cooperation, a gradual divergence of average values in the third and fourth waves of measurement can be observed, while for Responsibility the largest differences were recorded in the first wave of measurement.

**Table 10:** Differences in self-rating and teacher-rating depending on time and sex – social skills and their importance.

Social skills	*Wave*				Wave*sex			
*Self-rating*	*Wilk’s λ*	*F(3,292)*	*p*	*η*_*p*_^*2*^	*Wilk’s λ*	*F(3,292)*	*p*	*η*_*p*_^*2*^
Communication	0.99	0.96	0.414	0.01	0.98	2.47	0.062	0.03
Cooperation	1.00	0.52	0.672	0.01	0.99	0.97	0.410	0.01
Assertion	1.00	0.53	0.660	0.01	0.99	1.31	0.271	0.01
Responsibility	0.99	1.28	0.280	0.01	0.99	0.71	0.547	0.01
Empathy	0.99	1.39	0.247	0.01	0.98	1.59	0.192	0.02
Engagement	0.95	4.87	0.003	**0.05**	0.98	2.07	0.105	0.02
Self-Control	0.95	4.84	0.003	**0.05**	0.99	1.06	0.365	0.01
*Teacher-rating*								
Communication	0.98	2.51	0.059	0.03	0.99	0.88	0.454	0.01
Cooperation	0.99	1.31	0.272	0.01	0.97	2.68	0.047	0.03
Assertion	1.00	0.21	0.889	0.00	1.00	0.13	0.944	0.00
Responsibility	0.99	0.88	0.450	0.01	0.97	2.82	0.039	**0.03**
Empathy	0.97	2.67	0.048	**0.03**	0.98	2.52	0.058	0.03
Engagement	0.99	0.97	0.409	0.01	1.00	0.37	0.776	0.00
Self-Control	0.98	1.78	0.152	0.02	0.99	0.74	0.531	0.01
**Importance**	***Wave***				Wave*sex			
***Self-rating***	***Wilk’s λ***	***F(3,292)***	***p***	***η*** **_*p*_** **^*2*^**	***Wilk’s λ***	***F(3,292)***	***p***	***η*** **_*p*_** **^*2*^**
Communication	0.94	6.60	0.000	**0.06**	0.98	1.65	0.179	0.02
Cooperation	0.88	13.93	0.000	**0.13**	0.99	0.98	0.401	0.01
Assertion	0.94	5.91	0.000	**0.06**	1.00	0.21	0.896	0.00
Responsibility	0.87	14.41	0.000	**0.13**	0.98	1.66	0.176	0.02
Empathy	0.83	19.76	0.000	**0.17**	0.99	1.32	0.269	0.01
Engagement	0.90	11.14	0.000	**0.10**	0.99	1.48	0.221	0.02
Self-Control	0.96	4.56	0.004	**0.05**	0.99	1.47	0.224	0.02
*Teacher-rating*								
Communication	0.89	11.90	0.000	**0.11**	0.97	3.20	0.024	0.03
Cooperation	0.98	2.51	0.059	0.03	1.00	0.52	0.666	0.01
Assertion	0.98	2.13	0.096	0.02	0.98	2.37	0.071	0.02
Responsibility	0.93	7.52	0.000	**0.07**	0.97	3.57	0.015	**0.04**
Empathy	0.94	6.23	0.000	**0.06**	0.98	1.75	0.157	0.02
Engagement	0.89	11.78	0.000	**0.11**	0.98	2.10	0.101	0.02
Self-Control	0.99	1.12	0.339	0.01	0.99	0.85	0.467	0.01

Statistically significant statistics (*p* < 0.05) are bolded. Due to a violation of the sphericity assumption, the results of Multivariate Tests are interpreted instead of Tests of Within-Subjects Effects.

**Fig 3:**
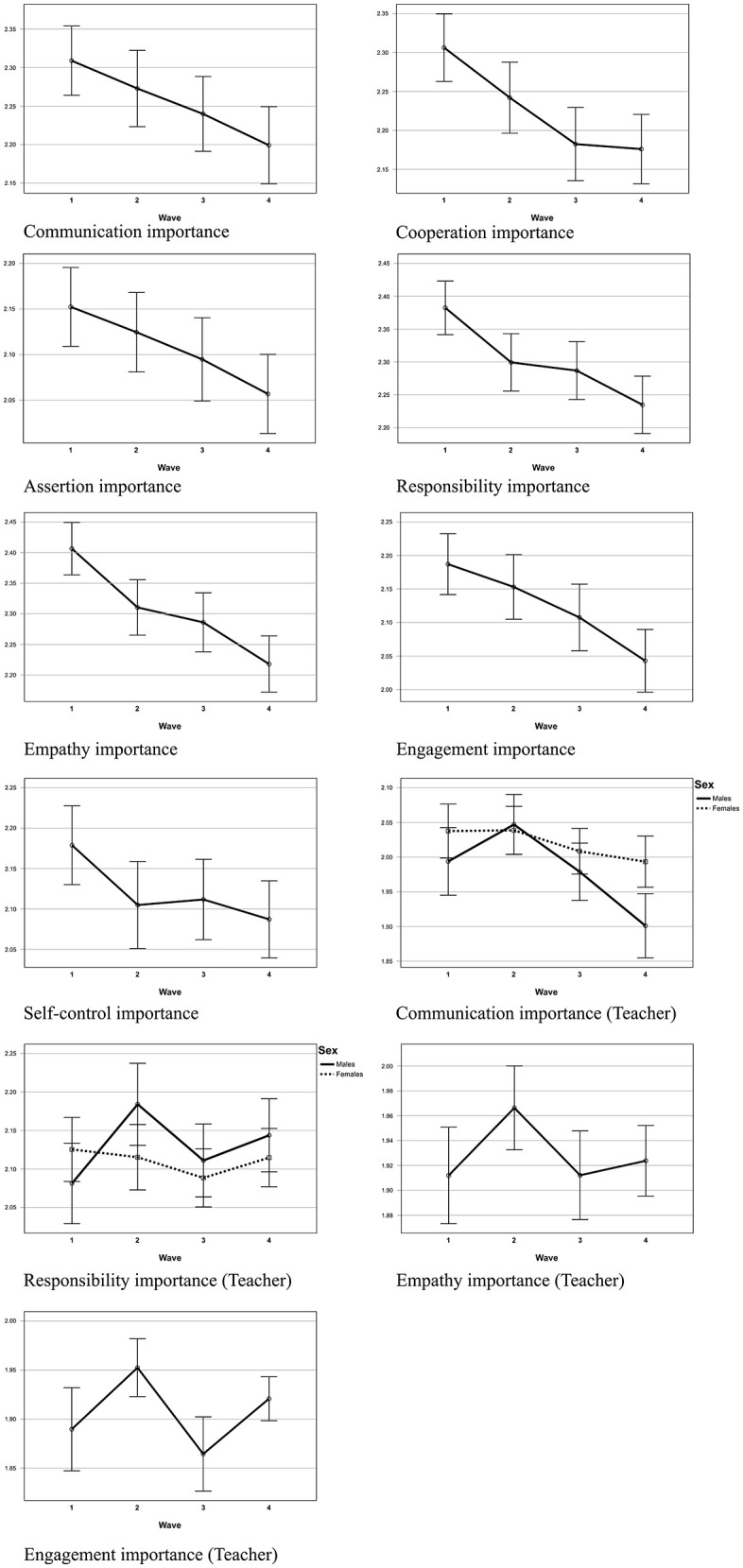
Differences in self-rating and teacher-rating are displayed based on time and sex – social skills importance. Note. Estimated marginal means and corresponding 95% confidence intervals are shown.

From the perspective of students, the importance of all social skills decreases over time (Fig 3). The low agreement between self-ratings and teacher ratings on the importance of social skills (see Table 6) is also reflected in the fact that the developmental trends captured in the self-ratings of students do not correspond to those of teacher ratings.

## Discussion

### Agreement between self-ratings and teacher ratings – personality traits

The findings of this study are consistent with previous research, as the highest agreement between self- and other-ratings of personality traits was found for Extraversion, a trait high in observability and low in evaluativeness, while the lowest agreement was found for Neuroticism, a trait with the opposite valence (low in observability and high in evaluativeness). Agreement between self- and teacher ratings was also found for traits that are manifested specifically in academic and learning behaviors, i.e., Openness to Experience and Conscientiousness [21], with a higher agreement for Conscientiousness found for girls than for boys. However, the agreement between the self-ratings and teacher ratings is only low to moderate in magnitude. Similar patterns of relationships were observed in all four measurement waves of the longitudinal study.

Beyond trait observability, the relatively modest magnitude of agreement may also reflect systematic sources of rater disagreement. One important factor is method variance associated with differences in assessment formats and perspectives. Self-reports capture internal experiences, self-concepts, and context-generalized tendencies, whereas teacher ratings are based primarily on observable behavior. These methodological differences can introduce systematic discrepancies that are independent of the traits themselves and may contribute to lower cross-informant agreement, as discussed, for example, by De Los Reyes [9] in the context of mental health assessment. As a result, disagreement between informants may partly reflect genuine cross-situational variability rather than measurement inaccuracy.

### Agreement between self-ratings and teacher ratings – social competencies

The agreement between self-ratings and teacher ratings on social competencies was not high and was weaker than for personality traits. In contrast to the analysis of personality traits, we observed differences according to the sex of the respondents. For boys, agreement between self- and teacher ratings was only observed for the social competence Cooperation, while more significant relationships were found for girls. Slightly higher agreement was observed between self- and teacher ratings for girls across the four measurement waves. In all cases, these were social competencies that are highly observable, which is consistent with findings from personality trait research. The lowest agreement between self- and teacher-ratings for both boys and girls was for Empathy, which is a social competence low in observability. These findings are consistent with the study by Mudarra et al. [22], who found agreement between self-ratings and teacher ratings only for Cooperation in a sex-undifferentiated cohort of students. One reason for the lower agreement between self-ratings and teacher ratings for boys could be that 75% of the teachers in our sample were females. According to Mullola et al. [23] male teachers perceived boys more positively and more capable in educational competence and teachability than female teachers.

Discrepancies in the assessment of social competencies may also be influenced by the relational context between teachers and students. The quality of the teacher–student relationship can shape both the amount and type of information available to the teacher, as well as potential biases in perception. Teachers who have more frequent, positive, or closer interactions with students may be better able to detect subtle aspects of their social functioning, whereas more distant or conflictual relationships may limit insight or introduce evaluative biases. Such relational factors may therefore contribute to variability in agreement across competencies and between students. Furthermore, social competencies are inherently context-dependent and may be expressed differently across social environments. Behaviors such as empathy or communication may manifest differently in peer interactions outside the classroom than in teacher-supervised settings. Consequently, discrepancies between self- and teacher ratings may partly reflect context-specific variations in behavioral expression, not disagreement regarding the same underlying construct.

### Stability of self-ratings and teacher ratings – personality traits

The stability of the rating of personality traits was generally very high in the period under study, which is consistent with current findings [24], from the perspectives of both students and teachers. However, a comparison of the stability coefficients shows that the teachers’ rating of the personality stability of students was higher than the students’ own rating of stability.

Teachers can be more objective in assessing personality than students themselves, because they can draw on years of experience with many different children. They are also able to identify age-appropriate and developmentally typical behavior and observe children’s social interactions in a structured environment [25]. On the other hand, teacher assessment may also be biased by number of factors, such as implicit norms and theories and individual preferences [26]. Teachers may also form a view of a student that they subsequently apply regardless of the student’s development and personality changes. Thus, a teacher’s rating of high student personality stability may reflect not only the degree of stability of the student’s personality, but also the teacher’s perception of the student.

### Stability of self-ratings and teacher ratings – social competencies

The temporal stability of the level of social competence was high from the perspective of both students and teachers and was generally comparable across different social skills regardless of the student’s sex. The findings therefore support the view that social competence is formed in early and middle childhood and remains stable from early adolescence onwards [14].

In the assessments of teachers, girls had higher levels of Cooperation and Responsibility. The higher level of social competencies of girls compared to boys has been documented by several studies [e.g., 13–15]. However, certain gender stereotypes may play a role in teacher ratings. Teachers’ implicit theories of social behavior are more negative for boys, with whom they associate externalizing behavior [23,27]. As a consequence, teachers may also perceive boys to be less responsible and cooperative.

### Agreement between and the stability of self-ratings and teacher ratings – importance of social competencies

Agreement between student and teacher ratings of the importance of social competencies has not been widely explored. Our findings revealed virtually no agreement between the self-ratings and teacher ratings of the importance of social competencies. This confirms our assumption that information that is only accessible to the self and not expressed in overt behavior is difficult for an outside observer to recognize and evaluate, unless the observer is someone close [2,6].

At the same time, this near-zero agreement may also be influenced by methodological factors similar to those discussed for the aforementioned characteristics. Among contextual factors that may further attenuate agreement, individual values, motivations, and situational priorities are likely to play an important role, as they may differ substantially between students and teachers. While students’ ratings may reflect personal goals and peer-related concerns, teacher ratings may be anchored in educational expectations and classroom norms. These differences in evaluative frameworks may further contribute to systematic divergence between informants.

The importance of all social competencies from the perspective of students decreased over time, which may be related to developmental trends in early adolescence. This period is characterized by the influence and importance of peers beginning to outweigh that of the student’s family. Thus, many adolescents succumb to peer pressure, which can also lead to negative consequences such as drinking, smoking, and sexual activity, and the diminishing importance of social norms and socially desirable behavior [28].

### Practical implications

Educational professionals should focus on actively identifying less conspicuous student manifestations, as the agreement between teacher ratings and students’ internal experiences is very low for traits with low observability, such as neuroticism or empathy. While teachers easily recognize extraversion and external cooperation, students’ internal anxieties or subjective sensitivities often remain hidden. For effective early prevention of interpersonal and school problems, it is therefore essential to combine multiple sources of information—such as student self-report questionnaires—and not rely solely on external observation within the structured classroom environment.

Schools should implement mechanisms for the critical reflection of gender differences when assessing social competencies, as teachers (who are often female themselves) tend to rate girls as more responsible and cooperative, while boys may face negative bias. This discrepancy may stem from implicit theories and norms that associate boys with externalizing behavior, thereby undervaluing their actual skills. Targeted teacher training regarding these biases can prevent situations where boys unnecessarily fail to meet standards that are primarily set based on the manifestations of girls.

School administrations and psychologists should address the risk of labeling students, which arises from the fact that teachers perceive students’ personality stability as higher than the students do themselves. Teachers may form a fixed image of a student and subsequently apply it regardless of the student’s actual development and changes over time. It is therefore necessary to apply a formative approach to assessment and regularly revise the perspective on the student, especially during early adolescence.

## Limitations

The predominance of female teachers in our sample may have influenced the ratings of students’ social competencies, as these may be more accurately assessed in boys by male teachers. Moreover, the teachers in our study rated students four times in a period of two years and thus may have been overwhelmed by the number of student assessments. This may have led them to project perceptions they had formed of students at the beginning of the study into their ratings. Although this was a longitudinal study, the four measurements were taken over a relatively short period of two years, which may be too short a time to observe developmental trends in personality traits and social competencies.

An important methodological limitation concerns the internal consistency of the employed scales. While most measures demonstrated adequate levels of reliability across both assessment contexts (self-ratings and teacher ratings), several scales reached only borderline values. In such cases, the observed student–teacher agreement is likely to underestimate the true level of correspondence due to attenuation effects associated with measurement error (see [29]). Particular caution is warranted when interpreting agreement for Extraversion (with the lowest McDonald’s omega), which appeared to be the most readily observable personality trait from the teacher’s perspective. In light of this limitation, it is plausible that Extraversion is, in fact, even more distinct from the other personality characteristics in terms of student–teacher agreement than the present findings indicate.

Our findings may also be influenced by the cultural context. Although the Czech Republic, as an active member of the OECD, has long integrated international standards into its educational policies, as evidenced by its regular participation in prestigious comparative studies such as PISA and TALIS, the teacher-student relationship in the Czech Republic is traditionally asymmetrical, emphasizing formal authority and external discipline—if a student’s internal anxiety does not disrupt the lesson and manifests quietly, it often remains invisible to the teacher.

## Conclusions

Current knowledge suggests that personal characteristics, whether personality traits or social competencies, that are manifested in behavior are more easily recognized by an external observer than characteristics that are not manifested in behavior and are only available to the Self. Teachers should be aware of this fact in the exercise of their profession and not rely only on what they can infer from the visible behavior of the students but also bear in mind that many of the personality characteristics of the child may be hidden to them. High levels of neuroticism can potentially lead to the development of internalized psychopathology (depression, anxiety), which is also more difficult for an outside observer to assess than externalized psychopathology [30]. Attention to hidden, unrevealed, or difficult-to-assess student characteristics may thus prevent developmental, school, and broader social/interpersonal problems in adolescents.

There are two main avenues for further research. First and foremost, our findings should be validated through a broader international study that would include not only OECD member countries but also countries from other cultural contexts. Further research should also be supplemented by a qualitative study following up on the questionnaire survey, which would examine the main sources of information about others from which raters draw their conclusions about their personalities, and would also identify typical ways of thinking about others. Ready et al. [31] noted that when peers were asked to rate a target on difficult-to-judge traits, they tended to draw on their own personalities; the authors referred to this phenomenon as “self-based heuristics.” Understanding such judgment processes may help clarify why some student characteristics remain hidden from teachers and may support more accurate, balanced, and developmentally sensitive assessment practices in schools.

## Supporting information

S1 File Inclusivity in global research questionnaire. (DOCX)
